# Recurrent intra-articular osteoid osteoma of the hip after radiofrequency ablation: a case report and review of the literature

**DOI:** 10.4076/1757-1626-2-6439

**Published:** 2009-07-17

**Authors:** Nicolas Efstathopoulos, Georgios Sapkas, Fragiskos N Xypnitos, Ioannis Lazarettos, Demetrios Korres, Vassilios S Nikolaou

**Affiliations:** 1 2^nd^ Orthopaedic Department, Athens University, Konstantopoulion “Agia Olga” General HospitalAthens, P.C 15124Greece; 2 1^st^ Orthopaedic Department, Athens University, Konstantopoulion “Agia Olga” General HospitalAthens, P.C 15124Greece

## Abstract

We present a case of a 53-year-old woman with recurrent intra-articular osteoid osteoma of the hip 6 months after initial treatment with percutaneous radiofrequency ablation. En bloc surgical excision of the osteoid osteoma and prophylactic internal fixation for impending stress fracture was performed. The patient is pain free, has returned to normal function and there is no sign of recurrence at the one-year follow-up. Intraarticular osteoid osteoma, present a diagnostic challenge and often they are misdiagnosed. Minimally invasive ablation techniques can fail in significant percentage and then surgical excision with histological confirmation remains the definitive treatment of choice.

## Introduction

Osteoid osteoma (OO) is a relatively common skeletal lesion that accounts for approximately 12% of benign skeletal neoplasms. Bergstrand was the first who described the pathological findings in 1930 [1], however Jaffe was the first to recognize osteoid osteoma as a separate entity, in 1935, in a report of five cases. Since then, more than 1,000 cases have been reported in the literature, establishing it as a common benign lesion.

For physicians, most of OO no longer presents a diagnostic problem, because the clinical, radiologic and scintigraphic characteristics have been well described. The classic symptoms, however, may not always be present, or they can be misleading and altered, especially when the tumor is located intraarticularly. Because of the unusual clinical and radiological features of this entity, intraarticular osteoid osteoma (IAOO) is described as a diagnostic challenge [2].

There is general agreement in the literature that complete excision is the treatment of choice and that incomplete removal of the nidus leads to recurrence of symptoms [3]. Recently, minimally invasive techniques, such as computed tomography (CT)-guided core-drill excision, arthroscopic removal, cryoablation and thermoablation by laser or radiofrequency energy have emerged alternatives to the conventional surgical excision. However, the success of these methods ranges from 70–100% [4].

In this paper, we report a recurrent intraarticular osteoid osteoma of the hip after radiofrequency ablation. Furthermore, we review the literature on the clinical and radiological features of IAOO, the diagnostic challenges and therapeutic options.

## Case presentation

A 53-year-old Greek woman, referred to our clinic in September 2006, six months after her initial treatment with percutaneous radiofrequency ablation for IAOO of the right hip joint, reporting recurrent symptoms.

She had no relevant or significant medical history, and she reported right groin pain radiating to the thigh for the past 4 years. Her initial symptoms were an aching groin and thigh pain that insidiously worsened over several weeks. Daily activities worsen the symptoms, and she had significant night pain. She reported that aspirin and non–steroid anti–inflammatory medications had provided significant relief only in the early stages of this ailment, reducing the pain.

Before the correct diagnosis and treatment with radiofrequency ablation she had had incorrect diagnoses for a time period of approximately 3 years. The misdiagnoses included bursitis, tendonitis and muscle strain.

The patient referred to our clinic, 6 months after radiofrequency ablation, reporting recurrent symptoms similar to those experienced at the initial clinical presentation. She reported that the recurrence occurred in the second month after initial treatment. Physical examination revealed moderately reduced right hip range of motion in all planes, with end–range pain in all directions. There was mild, antalgic, Trendelenburg gait. Neurologic examination found no abnormalities. Blood count and biochemical profile were within the reference ranges.

**Figure 1.**
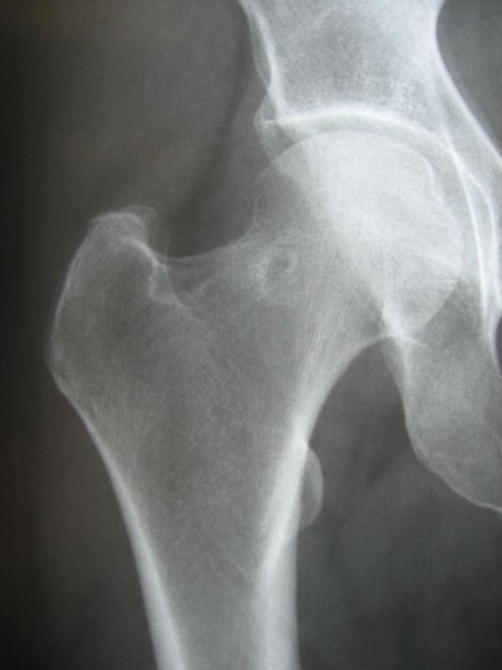
Plain radiograph showing an oval nidus surrounded by a radiolucent ring at the femoral neck proximal to the femoral head of the right hip 6 months after RF ablation.

**Figure 2.**
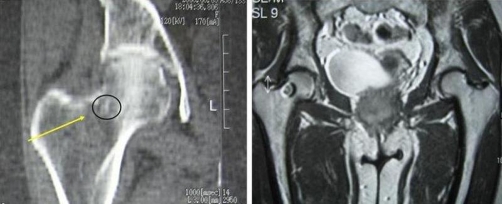
The CT representation of the lesion 3 months after RF ablation (left). The MRI representation of the lesion before RF ablation (right).

The radiograph showed an oval nidus surrounded by a radiolucent ring at the femoral neck proximal to the femoral head (Figure 1). Computed tomography and magnetic resonance imaging of the right hip joint revealed a lesion at the posterior cortex of the femoral neck, which was attributed to the previous radiofrequency ablation. They also revealed increased sclerosis at the posterior aspect of the femoral head with a central lucency. The lesion was located intramedullary and was smaller in diameter (0.7 cm) than the one that was revealed in the MRI before radiofrequency ablation (1.5 cm) (Figure 2).

**Figure 3.**
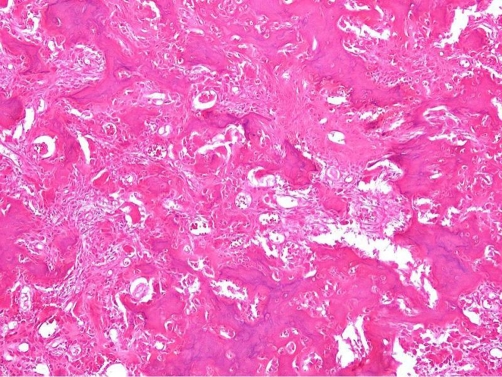
Histopathologic features of osteoid osteoma from the biopsy. Nidus displaying well mineralized trabeculae of woven bone lined by numerous active osteoblasts and multinucleated giant cell-like osteoclasts. Hematoxylin and eosin (X100).

We proceeded to an en bloc surgical excision of the lesion after patient’s denial to undergo a second percutaneous radiofrequency ablation. At surgery, the patient received general anesthesia. The operation was performed on a fracture table, under fluoroscopic control. An anterolateral approach to the hip joint was undertaken and the femoral neck region was exposed. Localization of the lesion, intra-operatively, was achieved with the use of two Kirschner wires under fluoroscopy. Small osteotomes, a high-speed burr and curettage were used to completely remove the lesion. Prophylactic internal fixation and grafting for impending stress fracture was performed. A three-hole DHS fixation was selected to allow for faster patient’s mobilization. Histological examination of the resected tissue, confirmed the diagnosis of OO (Figure 3). Normal bone histology from the adjacent area of the OO confirming the complete excision of the lesion.

**Figure 4.**
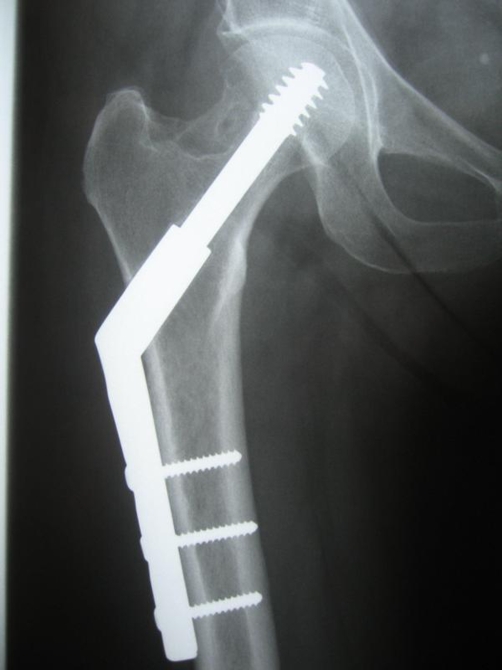
Plain radiography one-year post-operatively.

Post-operatively full weight bearing as tolerated was allowed. Within one month the patient had complete pain relief, had stopped using all medications, and had returned to her previous level of physical activity. At the one–year follow–up there were no residual or recurrent symptoms at the clinical or radiological findings (Figure 4).

## Discussion

Osteoid osteoma is a benign bone-forming tumor that involves cortical or cancellous bone. The lesion is usually less than 1.5 cm in size [2].

The lesion may occur in any bone, although there is a predilection for the lower extremity, with 50% or more of lesions occurring in the femur and tibia [5]. Pain is the leading symptom of the lesion. It is usually described as mild and intermittent at first, later becoming more constant and severe. Before the lesion is apparent radiologically, in some cases, intense pain may be present. Pain is frequently worse at night and may awaken the patient from sleep and often is relieved by aspirin or nonsteroidal anti-inflammatory drugs [4].

The imaging modality of choice for identifying the nidus of an OO is the CT. A CT scan usually gives a sharp contrast between the nidus and the surrounding normal trabecular bone even in the absence of a sclerotic rim. Rarely, CT results may be falsely negative. On the other hand, MRI is less useful than CT, and may miss small lesions, or show nonspecific changes [6]. Further, soft tissue and bone edema around the nidus can be confusing and can suggest an aggressive or malignant process.

Initial treatment of OO involves the use of NSAIDs or aspirin [7]. Complete surgical en bloc excision of the OO nidus is curative and brings immediate and dramatic relief of symptoms, and is still the preferred treatment method [8]. The clinical success rate of surgery ranges from 88 to 100% [9]. Nevertheless, alternative, less invasive methods, such as the percutaneous CT-guided RF ablation, are gaining popularity. Clinical success with these methods varies between 70-100% [4].

In the case of intraarticular location the clinical signs differ significantly from the well known classical symptoms of extraarticular lesion [6]. The most common symptoms that patients with IAOO complain for are articular pain, joint tenderness and effusion, soft-tissue swelling, stiffness and decreased ROM [10]. These lesions present a rate of approximately 13% and they are most commonly encountered in the hip. Small series have been reported in other joints such as the elbow the talus , the carpal joints the spine and the foot joins [2], however the majority of these IAOO osteomas have been described as case reports.

In contrast to the more common cortical OO, IAOO (cancellous and subperiosteal types) present a far more formidable diagnostic challenge to the orthopaedic surgeon. The intensity of the sclerosis around the nidus depends on the anatomic location of the lesion, so it is intensive in the diaphysis of a long bone and only mild in the substance of an epimetaphyseal trabecular bone. Therefore, intraarticular osteoid osteoma in conventional radiographs present little or no reactive sclerosis [6]. The nidus is detected in fewer than 50% of cases when it is below 3 mm in size. The joint space may be widened secondary to synovitis and joint effusion. In the hip, regional osteoporosis may also be present [11], and in patients with symptoms for at least 3 months it has been observed widening and foreshortening of the femoral neck and reduction in the height of the capital femoral epiphysis [11]. Due to functional differences between intra- and extracapsular periosteum there is a lack of extensive reactive sclerosis. The less pronounced sclerosis around an intraarticularly located nidus may also explain why bone scan shows increased uptake of tracer in the region of joints but it is nonspecific and often too diffuse for visualization of a nidus [12]. Therefore, used alone, it is less helpful in the differential diagnosis.

The nonspecific symptoms and the lack of characteristic radiological features of the IAOO frequently results in delays in diagnosis and treatment. Delays up to 2.5-3.5 years in the diagnosis and treatment can be reported [2]. In our case, the patient was misdiagnosed for a time period of about 3 years. The pathologic entities that should be taken under consideration in differential diagnosis include synovitis, early osteoarthritis, monoarticular rheumatoid arthritis, inflammatory arthritis, tuberculous arthritis, septic arthritis, Legg-Calvé-Perthes disease, Brodie’s abscess and intracortical chondroblastoma [13,14].

Difficulties with lesion access and localization of the intra-articular OO at surgery has made the newer minimally invasive interventions more appealing methods of treatment. Such methods include use of small instruments, resulting in a less invasive surgical approach and removal of less bone and also are reported to reduce cost, hospital stays and recovery time. However, there is still controversy regarding their success rates. It is well known that recurrence of OO is likely due to incomplete excision [15]. Especially recurrence after RF ablation has been reported to occur in up to 24% of patients, and most of them in the first 7 months after treatment [9]. In our case, the patient reported that the recurrence of symptoms occurred in the second month after her initial treatment with RF ablation. Eventhough secondary intervention with RF ablation can be more successful, failure to completely remove the lesion has been reported to a significant percentage of patients as well [9]. In these cases, surgery remains the standard treatment of choice.

## Conclusions

In conclusion, significant differences exist in the clinical and imaging features between intra- and extraarticular OO. IAOO present a diagnostic challenge and often they are misdiagnosed. Surgeons must be aware that in such cases careful approach with the use of the right diagnostic tools has to be enabled in order to avoid delays in the diagnosis and treatment. Finally, especially in these cases, minimally invasive techniques can fail in significant percentage and then surgical excision with histological confirmation remains the definitive treatment of choice.

## Abbreviations

CT: computed tomography
IAOO: intraarticular osteoid osteoma
NSAIDs: Non-steroidal anti-inflammatory drugs
OO: osteoid osteoma
RF: radiofrequency
